# Traumatic Dislodgement of Tibial Polyethylene Insert after a High-Flex Posterior-Stabilized Total Knee Replacement

**DOI:** 10.1155/2015/810716

**Published:** 2015-09-20

**Authors:** Juan Felix Astoul Bonorino, Pablo Ariel Isidoro Slullitel, Gonzalo Rodrigo Kido, Santiago Bongiovanni, Renato Vestri, Lisandro Carbó

**Affiliations:** The Knee Surgery Unit, Institute of Orthopaedics “Carlos E. Ottolenghi”, Italian Hospital of Buenos Aires, Potosí 4247, C1199ACK Buenos Aires, Argentina

## Abstract

Many pathologic entities can produce a painful total knee replacement (TKR) that may lead to potential prosthetic failure. Polyethylene insert dissociation from the tibial baseplate has been described most frequently after mobile-bearing and cruciate-retaining TKRs. However, only 3 tibial insert dislocations in primary fixed-bearing High-Flex posterior-stabilized TKRs have been reported. We present a new case of tibial insert dislocation in a High-Flex model that shares similarities and differences with the cases reported, facilitating the analysis of the potential causes, which still remain undefined.

## 1. Introduction

Although total knee replacement (TKR) is one of the most efficacious orthopaedic procedures, dissatisfaction and moderate pain after TKR are about 13% at one year [1] and 20.5% at two to seven years after surgery [2, 3]. Many pathologic entities can produce a painful TKR that may lead to potential prosthetic failure. Dissociation of the polyethylene tibial insert appears as a rare complication, which has been observed mostly in mobile-bearing implants. In recent years the number of cases reported in the literature is on the rise, although it is scarcely in fixed-bearing prosthesis. Therefore, it is necessary to be aware of the management of a patient with a possible insert dislocation.

## 2. Case Report

A 60-year-old man (height, 163 cm; weight, 80 kg; BMI, 30.1) with right knee osteoarthritis grade 3 of Kellgren-Lawrence classification [4] underwent a right TKR using a High-Flex, posterior-stabilized (PS), and fixed-bearing Exactech Optetrak High-Flex prosthesis (Miami, Florida, USA). Metal components were cobalt-chromium based alloys. A conventional medial para-patellar approach was performed and all components were fixed with cement. The patella was not resurfaced. Adequate ligamentous balancing as well as flexion and extension gaps were assessed intraoperatively and confirmed by postoperative standing radiographic control. As routinely, medial and lateral facets of the tibial baseplate were tested during surgery to corroborate complete locking of the posterior dovetails.

The rehabilitation protocol consisted of full-weight bearing and walker-assisted ambulation for a month and afterwards without any assistive device. The postoperative course was uneventful. There were neither popping sounds nor clicking sensations. An acceptable range of motion of 0°–130° was assessed at six months postoperatively, achieving 90 points of the International Knee Documentation Committee (IKDC) score.

**Figure 1:**
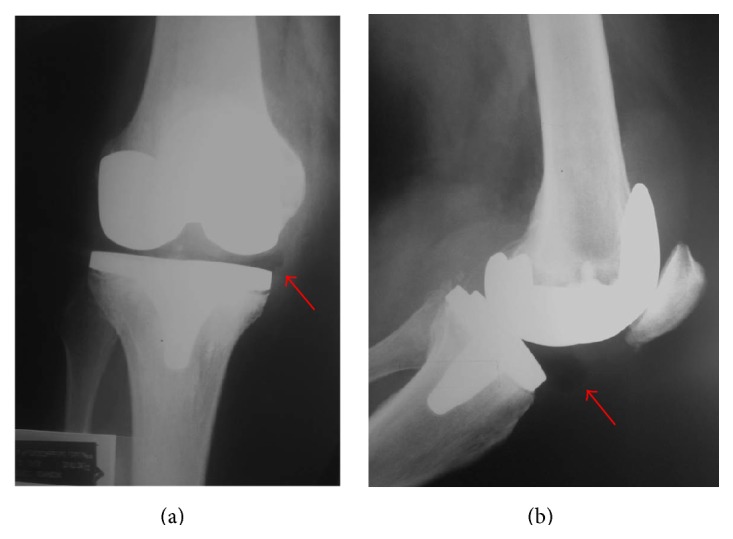
Anteroposterior (a) and lateral (b) radiographs of the right knee demonstrating a sign of anterior tibial insert dislocation.

At 32 weeks postoperatively, the patient complained of a sudden onset of knee pain associated with restricted range of motion. During physical examination, an audible popping sound was emanated from a swollen right knee. No signs of patellar instability were found. Although no specific injury was reported, the patient referred that a week earlier he had suffered a half-meter fall from a bus with both knees held in semiflexion. Radiographs evidenced a subtle sign of a polyethylene tibial insert dislocation, apparently displaced anteromedially (Figure 1). A CT-scan discarded component malrotation and loosening.

**Figure 2:**
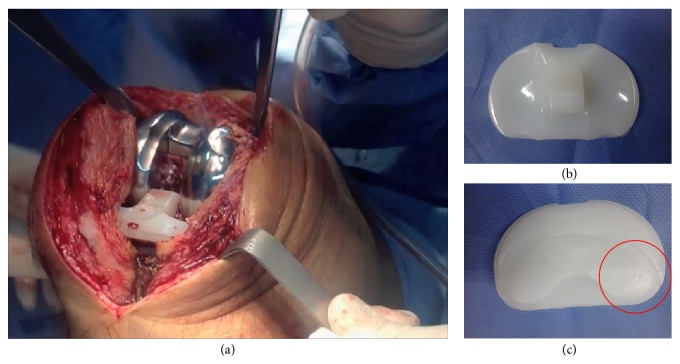
Intraoperative image (a) showing tibial insert dislodgment after an anterior approach of the right knee. Images of the superior (b) and inferior (c) aspects of the retrieved tibial polyethylene evidencing indemnity of the former and damage at the posteromedial zone of the latter one.

Surgery was undertaken to change the polyethylene insert. Although radiographic images had suggested a potential insert dislocation, an initial arthroscopy was performed to confirm the diagnosis and to discard other additional causes of painful TKR with restricted motion: patellar dislocation; patellar clunk syndrome; arthrofibrosis; loose bodies; and so forth. Open surgery was then executed following the same anterior approach, as shown in Supplementary Video 1 (in Supplementary Material available online at http://dx.doi.org/10.1155/2015/810716). The insert was found to be totally dislodged from the tibial baseplate and displaced anteriorly, as shown in Figure 2 and in Video 1. Macroscopic examination of the retrieved liner revealed compression deformation damage at the posteromedial aspect of the inferior surface of the polyethylene, keeping the superior face intact (Figure 2) (Video 1). Intraoperative C-reactive protein levels [5] and culture samples taken during surgery resulted negative for infection.

Femoral and tibial metal components were well fixed to bone and exhibited neither damage nor rotational deviation during intraoperative evaluation. Hence, a new 9-mm High-Flex PS liner was placed, immediately regaining a range of 0°–130°.

One month following the bearing exchange, the patient developed a postoperative infection secondary to a methicillin-resistant* Staphylococcus aureus*, which ultimately required a two-stage revision surgery.

## 3. Discussion

A recent study [6] concluded that the most common indications for revision TKR, besides aseptic loosening (21.8%), were instability (21.8%), malalignment (20.7%), and periprosthetic infection (14.5%). The authors have found a substantial reduction in implant-associated revisions, such as those related to polyethylene wear, due to improvements in implant performance and polyethylene manufacturing. Instability and malalignment may rarely result in a tibial insert dislocation. Nevertheless, many other factors have been associated with this event. Hence, the purpose of the case we report, besides describing it, was to analyze the underlying causes of insert dislodgement.

Polyethylene insert dissociation from the tibial baseplate has been described most frequently after mobile-bearing and cruciate-retaining (CR) TKRs [7, 8]. As stated by Thompson et al., the incidence of this event in mobile-bearing prosthesis oscillates between 0.4% and 9.3% [9]. However, only 3 tibial insert dislocations in primary fixed-bearing High-Flex PS TKRs have been reported. They all used Smith & Nephew Genesis II PS High Flex (Memphis, TEN, USA), similar to the case we report.

The patient reported by Rutten and Janssen [10] presented a direct trauma 14 months after primary surgery. The only intraoperative finding was a macroscopic damage of the polyethylene over its posterior surface, proximate to an osteophyte that remained posterior to the distal femur. This osseous structure may have progressively impinged the TKR leading to an eventual dislocation of the liner. In et al. [11] reported spontaneous, recurrent polyethylene insert dissociation after performing a mini-subvastus approach. They found a damaged tibial post that might probably lead to a pivot mechanism during maximum flexion. They said also that their limited incision with diminished field of view could have correlated with outcome. Finally, Lee et al. [12] presented a case of a spontaneous, belated polyethylene displacement at 2 years postoperatively, without prior trauma. They found an incomplete seating of the insert attributing the cause to the prosthesis design with thin posterior dovetails.

The current case has some differences with the former ones. Unlike Rutten and Janssen [10], it was not a direct trauma but an indirect one that could have originated the disengagement. Different to In et al. [11], no tibial post disruption was identified; and in contrast to Lee et al. [12], the time interval between the initial surgery and the onset of symptoms was considerably shorter. Nonetheless, all cases have in common damage at the posterior lips (medial and/or lateral) of the polyethylene's inferior surface as well as anterior displacement. These findings evidence failure at the posterior locking mechanism, during initial surgery or overtime. This is not a minor finding, since failure of the locking mechanism has been associated with the onset of osteolysis by generating backside wear and synovitis [13].

Additionally, it is not well defined whether this failure may lead to a definitive damage to the locking tab. Tradonsky et al. [14] tested the push-out strength necessary to dissociate the liner from eight acetabular cups in an in vitro study. Repetitive testing on the same acetabular component with a new polyethylene revealed progressive miscarriage of the locking mechanism. Similarly, Anderson et al. [15] described a case of recurrent tibial insert dislodgment in an obese (BMI 38) truck driver who continued to jump from heights, repeatedly challenging the tibial baseplate and polyethylene. They found that dislocation occurred with 2 different inserts on the same tibial baseplate, alleging that the anterior locking tab may have fatigued due to micromotion and previous dislodgement. Hence, they recommend revising the baseplate when there is a potential for new dislodgement or motion between the old baseplate and the new insert is encountered. As in the case we report, the patient was obese and the underlying cause of the insert luxation was an indirect trauma secondary to a jump.

Three potential risk factors could be distinguished from this report and its bibliographic analysis, without any statistical value: error in the surgical technique, prosthesis design, and patient-related causes. Regarding the surgical technique, the next miscalculations should be contemplated: insufficient flexion-extension gap; ligamentous instability; and inadequate seating of the tibial insert on the baseplate's locking mechanism. Especially after mini-invasive approaches, insufficient visualization for insertion of the polyethylene liner may not be available leading to incomplete seating of it on the tibial baseplate.

When using mobile-bearing designs, contact between the femoral component and the insert should be smooth, in a perfectly matched position. When the femoral component is put slightly posterior compared to a matched position, trivial knee flexion results in downward force to the posterior aspect of the polyethylene, producing an anterior lift-off by a pivoting effect made throughout the locking ring [7]. Analyzing bearing dislocations in CR models, Fisher et al. [8] assumed that the spinout mechanism might occur in a semiflexed adducted knee by causing a lateral compartment opening. This would allow the lateral femoral condyle to escape over the anterior lip of the insert and then fall-off in front of the bearing, forcing it to externally rotate and fail. The Optetrak High-Flex design has got some characteristics that might have contributed to the dissociation. Unlike other models, the femoral guide is not designed to additionally cut the posterior femoral condyles so as to increase the femoral posterior translation and allow for maximum flexion. Thus, the possibility of reaching maximum flexion would be demanding, as pressure would increase on the posterior dovetails and on the tibial post. Additionally, we believe that the anterior tab of the liner is quite thin [10]. This shallowness would promote an anterior lift-off over the metal tray if the insert was incompletely seated or deformed.

And as for patient-related causes, these should be highlighted: young active patients; increased BMI; and high-impact activities such as jumping. Though not completely understood, a flaw in the locking mechanism may be implied in patients with these characteristics, which might not be able to tolerate the increased demand at the modular junction.

Although extremely infrequent, tibial insert dislocation should be suspected when clicking or popping is associated with diminished range of motion along with knee swelling. Acute instability may sometimes be subtle. Radiographic analysis must be meticulous, searching initially for anterior liner subluxation. When diagnosis is still doubtful, a knee arthroscopy may prove useful. Since there are no strong evidence-based conclusions in the literature, the gold standard for treatment remains unknown. Some authors prefer only changing the polyethylene, whereas some others consider dissociations to enduringly incapacitate the locking mechanism of the tibial baseplate [14, 15], thus pondering the revision of the tibial baseplate.

## 4. Conclusion

In the reported case, the traumatic dissociation of the polyethylene may be associated with a jump from height with the knee held in semiflexion, which probably strained the insert by compression forces at the posteromedial aspect, damaging the locking mechanism. The High-Flex model may have contributed to the dislocation due to its unique design. The patient's high BMI should also be born as an adjunct.

## Supplementary Material

Video 1 shows intraoperative findings during the tibial insert exchange. Arthroscopy evidenced an anterior lift-off and complete dissociation of the polyethylene out of the tibial baseplate and discarded other additional causes of painful TKR. Open surgery revealed compression deformation damage at the posterior aspect of the inferior surface of the retrieved polyethylene.
